# Static and Dynamic Dysconnectivity in Early Psychosis: Relationship With Symptom Dimensions

**DOI:** 10.1093/schbul/sbae142

**Published:** 2024-08-30

**Authors:** Giulia Cattarinussi, David Antonio Grimaldi, Mohammad Hadi Aarabi, Fabio Sambataro

**Affiliations:** Department of Neuroscience (DNS), University of Padova, Padua, Italy; Padova Neuroscience Center, University of Padova, Padua, Italy; Department of Neuroscience (DNS), University of Padova, Padua, Italy; Department of Neuroscience (DNS), University of Padova, Padua, Italy; Padova Neuroscience Center, University of Padova, Padua, Italy; Department of Radiology and Medical Imaging, University of Virginia, Charlottesville, Virginia, USA; Department of Neuroscience (DNS), University of Padova, Padua, Italy; Padova Neuroscience Center, University of Padova, Padua, Italy

**Keywords:** functional magnetic resonance imaging, independent component analysis, dynamic functional network connectivity, psychosis

## Abstract

**Background and Hypothesis:**

Altered functional connectivity (FC) has been frequently reported in psychosis. Studying FC and its time-varying patterns in early-stage psychosis allows the investigation of the neural mechanisms of this disorder without the confounding effects of drug treatment or illness-related factors.

**Study Design:**

We employed resting-state functional magnetic resonance imaging (rs-fMRI) to explore FC in individuals with early psychosis (EP), who also underwent clinical and neuropsychological assessments. 96 EP and 56 demographically matched healthy controls (HC) from the Human Connectome Project for Early Psychosis database were included. Multivariate analyses using spatial group independent component analysis were used to compute static FC and dynamic functional network connectivity (dFNC). Partial correlations between FC measures and clinical and cognitive variables were performed to test brain-behavior associations.

**Study Results:**

Compared to HC, EP showed higher static FC in the striatum and temporal, frontal, and parietal cortex, as well as lower FC in the frontal, parietal, and occipital gyrus. We found a negative correlation in EP between cognitive function and FC in the right striatum FC (*p*_FWE_ = 0.009). All dFNC parameters, including dynamism and fluidity measures, were altered in EP, and positive symptoms were negatively correlated with the meta-state changes and the total distance (*p*_FWE_ = 0.040 and *p*_FWE_ = 0.049).

**Conclusions:**

Our findings support the view that psychosis is characterized from the early stages by complex alterations in intrinsic static and dynamic FC, that may ultimately result in positive symptoms and cognitive deficits.

## Introduction

Despite decades of research have helped us gaining insight into the pathophysiology of psychosis, the underlying neurobiology of psychotic symptoms remains still largely unclear.^1,2^ Against a long-standing tradition that tried to explain psychosis focusing on single brain areas, the dysconnectivity hypothesis claims a primary etiological role for abnormal functional integration between different brain regions.^3,4^ The functional integration can be characterized in terms of functional connectivity (FC), defined as the temporal dependence between remote areas.^5,6^ Resting-state functional magnetic resonance imaging (rs-fMRI) has been widely used to assess spontaneous brain activity and consequently, the FC in the human brain, revealing the presence of networks of anatomically separated regions that are strongly functionally linked during rest.^6^ Moreover, it allowed us to assess the temporal relationships between these networks, defined as functional network connectivity (FNC).^7^

In chronic psychosis, along with subcortico-cortical dysconnectivity,^8^ meta-analytic evidence suggests hypoconnectivity within networks, including the default mode network (DMN), salience network (SN), somatosensory network (SM), and cognitive control network (CC).^9,10^ Notably, the chronic effects of antipsychotic treatment are believed to influence the brain structure and function in patients with psychosis, along with other factors related to the disease, such as metabolic changes, chronic stress, smoking, and substance abuse.^11,12^ Moreover, well-established evidence shows that the disease process itself can contribute to progressive cognitive decline and brain changes from the earliest stages.^11^ Therefore, the assessment of early psychosis (EP), where the influence of these processes is limited, could play a pivotal role in elucidating the genuine neurobiology of the disease.^2,13^ Evidence supporting altered FC in EP is heterogeneous, although some consistent findings have been reported, such as the hypoconnectivity between the striatum and the cortical structures of the SN, including the anterior insula and the cingulate cortex.^2,14^ Moreover, Sarpal et al observed higher FC between the cingulate cortex and the striatum with clinical improvement of psychotic symptoms in EP.^15^ Notably, the functional dysconnectivity of the striatum and of other SN structures has been also linked to impaired cognitive performance in psychosis.^16^

Growing evidence reveals that the brain displays spontaneous fluctuations of activity and FC on a slow time scale, while most available studies measured the FC averaged over multiple minutes (the so-called static FC, sFC), blending different states and possibly accounting for the heterogeneity of the results.^17,18^ In contrast, dynamic functional network connectivity (dFNC) analyses allow the study of evolving FC patterns, in terms of the characteristics and persistence of reoccurring states and quantitative measures of the dynamism of FC patterns over time.^19,20^ Interestingly, dFNC changes seem to correspond to electrophysiological properties and to predict cognitive functioning in healthy adults;^21,22^ in addition, dFNC changes have been reported in many neurological and psychiatric disorders.^23–25^ In psychosis, dFNC analyses revealed alterations that occur only during certain dynamic states and thus unobservable with sFC.^18^ These include reduced dynamism,^20,26^ and a tendency to reduce dwell time in strongly connected states and increase dwell time in sparsely connected states.^17,18,26,27^ Notably, a contrasting pattern of reduced dwelling in sparsely connected states was shown in unmedicated patients.^28^ Importantly, literature on dFNC alterations in EP is still limited,^29,30^ resulting in a gap of knowledge about the changes over time of FC patterns in this populations.

In this study, we employed rs-fMRI to explore static and dynamic connectivity in a sample of individuals with EP, with an onset within the past 5 years prior to the study. We hypothesized that the application of dynamic FC analysis on a sample of patients minimally affected by characteristic progressive brain changes would provide significant insight into the neurobiological underpinnings of psychosis, revealing early changes that may go undetected in the sFC analysis. Specifically, we anticipated the predominance of strongly connected states, in accordance to the finding of Lottman et al on unmedicated patients,^28^ and reduced dynamic fluidity. Furthermore, we evaluated the relationship between FC metrics and a range of clinical, cognitive, and emotional variables. We hypothesized that a reduction in dynamic fluidity would be associated with an increase in psychotic symptoms and that static and dynamic parameters would have an influence on psychotic symptoms and cognition.

## Material and Methods

### Participants

Data were obtained from the online database Human Connectome Project for Early Psychosis Release 1.1 (https://www.humanconnectome.org/study/human-connectome-project-for-early-psychosis). In short, the study contains neuroimaging, psychopathological, and cognitive data of 183 outpatients with the diagnosis of schizophrenia spectrum disorders, major depressive disorder (MDD) with psychotic features or bipolar disorder (BD) with psychotic features with symptoms onset within 5 years prior to study entry, and 68 matched healthy controls (HC). The inclusion criteria for both groups were: (1) 16–35 years of age; (2) ability to provide informed consent; (3) ability to communicate in English. Exclusion criteria were: (1) active medical condition affecting the brain or cognitive functioning; (2) mental retardation; (3) contraindications to magnetic resonance imaging (MRI); (4) substance-induced psychosis or psychotic disorder due to a medical condition; (5) severe substance use disorder in the previous 90 days; (6) electroconvulsive therapy in the previous 12 months; (7) high suicide risk. HC had no lifetime or current BD, recurrent MDD, schizophrenia (SCZ), and other psychotic disorders, current anxiety disorder, no first-degree family member diagnosed with SCZ spectrum disorders, and no psychiatric medications at the time of study entry. Additionally, we excluded subjects with current alcohol (*n* = 6) or cannabis abuse (*n* = 16) and 2 subjects were excluded for not having completed the diagnostic interview. Of the remaining participants, subjects were excluded from the analysis due to the lack of structural imaging data (*n* = 3) and excessive head movement during the rs-fMRI scan based on the excluding criteria of 3.0 mm and 3.0° in maximum head motion (*n* = 6), which resulted in a final sample of 96 patients with EP and 56 HC.

### Clinical Assessment and Cognitive Measures

Diagnostic clinical interviews were conducted with the Structural Clinical Interview for DSM-5—Research Version (SCID-5-RV).^31^ Intellectual abilities were assessed with the WASI-II,^32,33^ cognitive functions with the NIH Toolbox Cognition,^34–36^ psychotic symptoms with the Positive and Negative Syndrome Scale (PANSS).^37^

### Imaging Acquisition

Images were acquired at 3 imaging sites (Brigham and Women’s Hospital, McLean Hospital, and Indiana University) with 3 Siemens MAGNETOM Prisma 3T scanners. Two sites employed a 32-channel head coil, and the other used a 64-channel head and neck coil, with the neck channels turned off. All protocols were based on the 2016 CCF template protocol (https://www.humanconnectome.org/hcp-protocols-ccf-template). Briefly, the protocol scan sequences were as follows: T1-weighted MPRAGE structural scans of 0.8 mm isotropic resolution (TR = 2400 ms; TE = 2.24 ms; flip angle = 8). and rs-fMRI of 2 mm isotropic resolution, multiband acceleration factor of 8, TR/TE = 720/37 ms, FOV = 208 mm; 72 slices; Flip angle = 52°; time points = 400 acquired with AP phase encoding. Subjects were asked to remain still during the scans. During the rs-fMRI subjects remained with their eyes open and were required to fixate a white cross on a dark background, to think of nothing in particular, and not to fall asleep.

### Image Processing

Structural and functional magnetic resonance imaging data were preprocessed using Data Processing & Analysis for Brain Imaging (DPABI, http://rfmri.org/dpabi) and Statistical Parametrical Mapping 12 (SPM12) (http://www.fil.ion.ucl.ac.uk), running under MATLAB R2016a (The Mathworks, Sherborn, MA, USA). Briefly, all images were reoriented and realigned for head motion correction. Then, the T1-weighted images were segmented into gray matter, white matter, and cerebrospinal fluid. Gray matter images were then normalized to the Montreal Neurological Institute (MNI) space using DARTEL registration with a resulting isotropic voxel size of 3 mm × 3 mm × 3 mm.

A group spatial independent component analysis (ICA) was carried out using GIFT (http://icatb.sourceforge.net) to extract independent components, consisting of spatial maps and time courses (TCs) and to evaluate FNC. A total of 53 ICs were identified by applying the Neuromark algorithm, belonging to subcortical (SC), auditory (AUD), sensorimotor (SM), visual (VIS), CC, default mode (DMN), and cerebellum (CB) networks.^38^ Spatial maps were thresholded with a *t*-score > mean + 4 standard deviations.^39^ For each component, the TCs were detrended (removing the mean, slope, and period π and 2π sine and cosine functions to avoid skewing in the averaging of the tapers) using the multi-taper method implemented in Chronux (http://chronux.org), with the time-bandwidth equal 3 and the number of tapers equal 5. After detrending, TCs were despiked using the median absolute deviation as in 3Ddespike (https://afni.nimh.nih.gov/pub/dist/doc/program_help/3dDespike.html) and low-pass filtered (fifth-order Butterworth with high-frequency cutoff = 0.15 Hz). TCs were then pairwise correlated, and Fisher’s *Z*-transformed, thus resulting in a 53 × 53 FNC cross-correlation matrix.

### Static Functional Network Connectivity

First, for each feature type, we created a design matrix with the following predictors: age, diagnosis (EP, HC), mean frame-wise displacement (FD), and root mean square of motion.^40,41^ Then, we used a backward step-wise multivariate selection approach within GIFT 3.0 toolbox (http://mialab.mrn.org/software/mancovan/index.html) to determine differences in SMs, spectra, and FNC between EP and HC. We first conducted a multivariate analysis of covariance to identify significant predictors (false discovery rate (FDR)^42^ with α = 0.05) within the design matrix and then used univariate analysis to test the significance of the reduced model relative to the full model to identify specific relationships between diagnosis and spatial maps, spectra, and FNC for each intrinsic network, while ruling out the effects of nuisance covariates. All coordinates are reported in MNI space.

### dFNC

The dFNC analysis was based on the TCs of the FC as derived from the previous analyses, and was conducted with a sliding window approach using the dFNC toolbox within GIFT 3.0.^20^ Rs-fMRI images were divided into windows of 30 repetition times (60 s) size, with a Gaussian window alpha value of 3, and regularized using a Graphical LASSO L1 algorithm with 10 repetitions. The window was slid stepwise by 1 TR. Head motion parameters were regressed out from the TCs on the sliding window correlation. To estimate dFNC, we used 2 different statistical approaches. The first, clustering state analysis, assesses the structure of reoccurring patterns as well as the frequency and duration of their presentation. To identify these cluster states in the native state space, we applied a *k*-means clustering (*k* = 5) in accordance with previous literature,^20,43,44^ which was repeated 100 times on windowed FC matrices. Then, the mean time dwelled in each state by each subject was estimated. The number of transitions (NT) between clusters during the scan provides an estimate of dynamism, although possibly underestimating it when multiple FC pattern changes occur within a single cluster and overestimating it when multiple minimal pattern changes occur between different clusters.^20,39^ The second approach, meta-states analysis, can overcome this problem.^20^ Each dFNC matrix is modeled as a weighted sum of a finite number of maximally independent states, obtained using spatial group ICA on group dFNC with a model order of 5, which is considered an adequate dimensionality to include complex additive effects and keeps a richly featured basis pattern.^20^ The resulting vectors, called meta-states, are used for the characterization of moving from 1 meta-state to another in a 5-dimensional space. The trajectories from 1 meta-state to another within this space are calculated for each subject at any time point. Here, the dynamism is reflected by measures of dynamic fluidity, including the number of meta-states and of meta-state changes, and measures of dynamic range, which include the span, ie, the largest city block distance between 2 meta-states, and the total distance of the meta-states, ie, the overall distance traveled through the 5-dimensional space. At an exploratory level, we repeated the analyses with a *k*-means clustering employing *k* = 4 and *k* = 6.

## Statistical Analyses

For clinical, demographic, cognitive, and imaging measures, we estimated differences between groups using a *t*-test, χ^2^, or Mann-Whitney test, as appropriate. Given our a priori hypothesis of reduced dynamism in psychosis,^20,26,45^ partial correlations were used to assess the correlation between meta-state parameters and positive symptoms, which are the hallmarks of psychotic disorders. Given the high inter-correlation between meta-state parameters, this analysis was corrected for multiple comparisons using a Bonferroni correction with the Dubey and Armitage-Parmar method.^46^ Additional exploratory partial correlation analyses were performed in EP to assess the relationship between clinical and cognitive scores and all parameters of sFC and dFNC, applying an unadjusted FWE correction at *P* = .05 for multiple comparisons. In these correlations, for those variables that were not normally distributed in the EP group (*P* < .05 on the Shapiro-Wilk test), we used Box-Cox transformations. Chlorpromazine equivalents, estimated with the Gardner approach,^47^ were included as nuisance variables in all correlation analyses to mitigate the confounding effect of antipsychotic treatment on the relationship between scores and FC parameters (already adjusted for age, mean FD, and root mean square of motion). Furthermore, between-group differences and brain-behavior correlations were assessed in antipsychotic-free subjects. All statistical analyses were performed with Jamovi 2.3.21.0^48^ and with R version 4.2.2.^49^

## Results

### Demographic, Clinical, and Cognitive Data

The sample included 96 EP (mean age = 22.8 ± 3.9 years, 58 males) and 56 HC (mean age = 24.8 ± 4.2 years, 37 males). No differences in sex (χ^2^ = 0.48, *P* = .49) were observed between the 2 groups, while age (*U* = 1896, *P* = .002) and IQ (Welch’s *t* = −5.4, *P* < .001) were significantly lower in EP compared to HC. Sociodemographic and clinical data are reported in table 1. Patients with EP performed significantly lower on the NIH Toolbox Cognition (*U* = 1020, *P* < .001), with a mean score of 100 ± 13.5 in EP and of 113 ± 8.1 in HC. In the EP group, 44 patients (45.8%) were taking antipsychotics and 52 (54.2%) were antipsychotics-free. Analyses excluding subjects treated with antipsychotics are reported in supplementary material. EP presented higher FD compared to HC (*P* = .036) (see supplementary material).

**Table 1.** Demographic and Clinical Variables

	EP (*n* = 96)	HC (*n* = 56)	*χ* ^2^ or *t* or U	*P*
Age [m (SD), ys]	22.83 ± 3.89	24.75 ± 4.15	*U* = 1896	.002
Sex [M/F]	58/38	37/19	*χ* ^2^ = 0.483	.487
Handedness (R/L/A)	82/9/4	45/10/1	*χ* ^2^ = 3.34	.343
Full IQ	102 ± 16.9	116 ± 10.6	*t* = −5.36	<.001
SES	2.25 ± 1.13	2.09 ± 1.06	*U* = 2423	.434
AP/non AP	44/52	—	—	—
AP exposure (months)	15.56 ± 16.16	—	—	—
AP daily dose, CPZ mg equivalents (m, SD)	165.78 ± 217	—	—	—

*Note*: A, ambidextrous; AP, antipsychotics; CPZ, chlorpromazine; EP, early psychosis; F, female; HC, healthy controls; IQ, intelligence quotient; L, left; m, mean; M, male; R, right; SD, standard deviation; SES, socio-economic status.

### Static Functional Network Connectivity

**Fig. 1.**
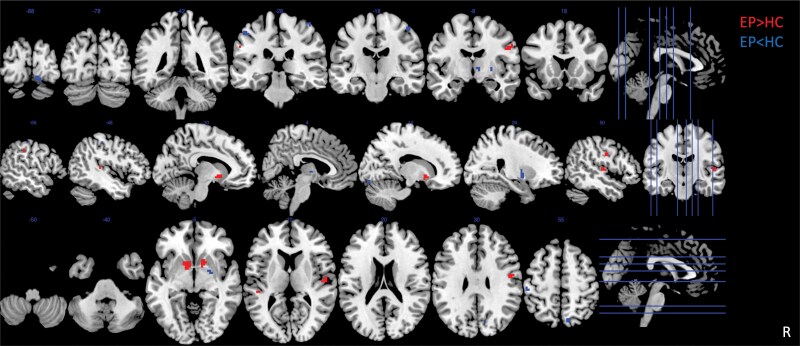
Intrinsic connectivity in EP compared to HC in the left (*x*,*y*,*z* = −12,9,−3) and right striatum (*x*,*y*,*z* = 18,9,−3) within the SC network, left (*x*,*y*,*z* = −48,−30,9) and right superior temporal gyrus (*x*,*y*,*z* = 48,−15,9), left superior frontal gyrus (*x*,*y*,*z* = −24,−3,66), left inferior parietal lobule (*x*,*y*,*z* = −54,−30,39), and right postcentral gyrus (*x*,*y*,*z* = 56,−27,45) within the SM network. Decreased connectivity in EP compared to HC in the right striatum (*x*,*y*,*z* = 21,0,−6) within the SC network, in the right SFG (*x*,*y*,*z* = 18,−12,69), left postcentral gyrus (*x*,*y*,*z* = −51,−24,−51), right precentral gyrus (*x*,*y*,*z* = 45,−21,51), and right precuneus (*x*,*y*,*z* = 9,−69,54) within the SM network, in the right precuneus (*x*,*y*,*z* = 15,−63,21) and left superior occipital gyrus (*x*,*y*,*z* = −12, −100,11) within the VIS network. Probability maps of intrinsic network loadings differences are thresholded at *P* = .005 and corrected for multiple comparisons with α = 0.05 and overlaid on the Montreal Neurological Institute brain template. The color bar indicates *t*-scores.

Multivariate analyses yielded significant differences in spatial maps between EP and HC. In particular, we found an effect of the diagnosis on the spatial maps of the subcortical (SC; IC1, IC4), auditory (AUD; IC 7), sensorimotor (SM; IC8–13, IC15–16), visual (VIS; IC17–21, IC23, IC24), executive (EXE; IC26, IC32), default mode network (DMN; IC47, IC48), and cerebellar (CB; IC51) networks (supplementary figure 1). Within the SC network, univariate analyses confirmed that the IC loadings were significantly increased in the left caudate/putamen (*x*,*y*,*z* = −12,9,−3; IC4) and right putamen (*x*,*y*,*z* = 18,9,−3; IC4) and decreased in the right globus pallidus (*x*,*y*,*z* = 21,0,−6; IC4) in EP compared to HC; within the SM network, the IC loadings were significantly increased in the left (*x*,*y*,*z* = −48,−30,9; IC8) and right superior temporal gyrus (STG) (*x*,*y*,*z* = 48,−15,9; IC8), left superior frontal gyrus (SFG) (*x*,*y*,*z* = −24,−3,66; IC16), left inferior parietal lobule (IPL) (*x*,*y*,*z* = −54,−30,39; IC16), and right postcentral gyrus (*x*,*y*,*z* = 56,−27,45; IC16) and reduced in the right SFG (*x*,*y*,*z* = 18,−12,69; IC10), left postcentral gyrus (*x*,*y*,*z* = −51,−24,−51; IC11), right precentral gyrus (*x*,*y*,*z* = 45,−21,51; IC11), and right precuneus (*x*,*y*,*z* = 9,−69,54; IC15). Lastly, within the VIS network, the IC loadings were reduced in the left superior occipital gyrus (SOG) (*x*,*y*,*z* = −12,−100,11; IC18) and the right precuneus (*x*,*y*,*z* = 15,−63,21; IC17; figure 1, table 2). The analyses on antipsychotic-free subjects confirmed all the results of medicated EP (see supplementary material).

**Table 2.** Static FC Parameters in EP and HC

	EP (*n* = 96)	HC (*n* = 56)	*t* or *U*	*P*
Left striatum	3.96 ± 0.71	3.33 ± 0.62	*t* = 5.48	<.001
Right striatum	5.78 ± 0.96	5.09 ± 0.69	Welch’s *t* = 5.11	<.001
Right striatum	3.48 ± 0.67	4.05 ± 0.67	*t* = −5.03	<.001
Left STG	1.44 ± 0.85	0.75 ± 0.73	*U* = 1442	<.001
Right STG	0.47 ± 0.53	0.04 ± 0.41	*t* = 5.31	<.001
Right SFG	0.79 ± 0.71	1.36 ± 0.77	*t* = −4.69	<.001
Left postcentral gyrus	1.98 ± 1.10	2.92 ± 1.40	*U* = 1557	<.001
Right precentral gyrus	7.83 ± 1.83	9.63 ± 1.94	*U* = 1277	<.001
Right precuneus	4.30 ± 1.62	5.68 ± 1.50	*U* = 1439	<.001
Left SFG	1.08 ± 0.99	0.53 ± 0.60	*U* = 1677	<.001
Left IPG	2.86 ± 1.12	2.09 ± 0.69	*U* = 1488	<.001
Right postcentral gyrus	3.83 ± 1.40	3.00 ± 1.17	*t* = 3.74	<.001
Left postcentral gyrus	2.10 ± 1.34	3.14 ± 1.61	*U* = 1677	<.001
Right precentral gyrus	1.69 ± 1.32	2.78 ± 1.35	*U* = 1440	<.001
Right precuneus	4.52 ± 1.28	5.58 ± 1.71	*U* = 1731	<.001
Left SOG	1.34 ± 1.04	2.24 ± 1.21	*t* = −4.87	<.001

*Note*: EP, early psychosis; HC, healthy controls; IPG, superior parietal gyrus; SFG, superior frontal gyrus; SOG, superior occipital gyrus; STG, superior temporal gyrus.

### dFNC

**Fig. 2.**
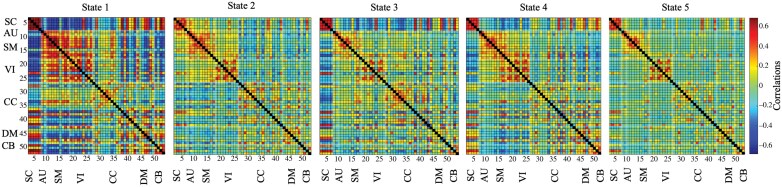
Cluster states resulting from the dFNC analysis were identified by *k*-means clustering (*k* = 5) on the whole-group level. Median cluster centroids in the correlation matrices for each of the 5 dFNC States are reported. The color bar indicates the magnitude of each correlation. SC, subcortical network; AU, auditory network; SM, sensorimotor network; VI, visual network; CC, cognitive control network; DM, default mode network; CB, cerebellum network.

**Fig. 3.**
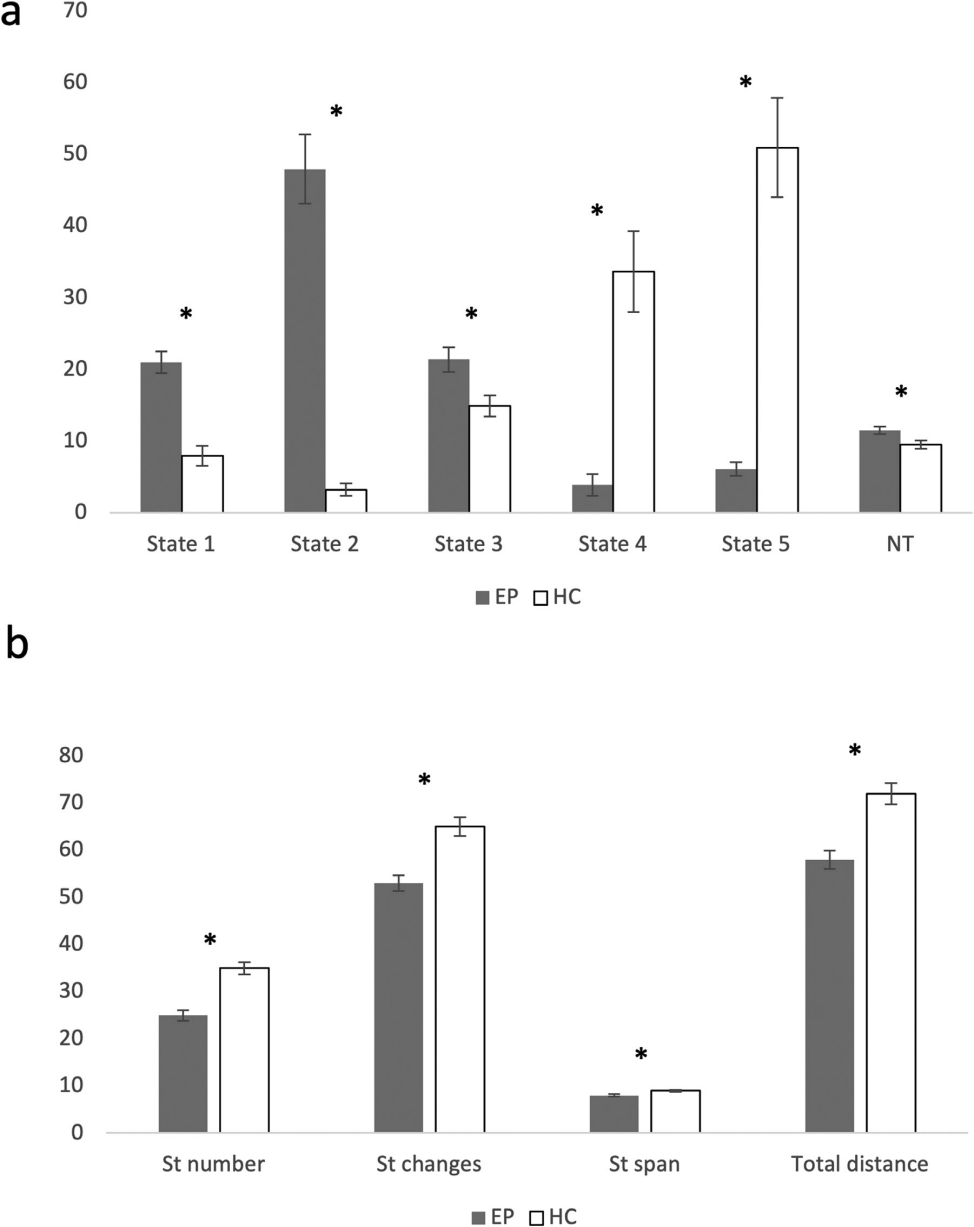
(a) Dwell times in the different state clusters and number of transitions between clusters in EP and HC with respective confidence intervals. EP is indicated in gray and HC in white. Dwell times are given in number of TR windows. All the differences are significant at *P* < .05. NT, number of transitions. (b) Number of meta-states, number of state changes, state span, and total distance, with respective standard errors. All the differences are significant at *P* < .05.

Among the 5 estimated cluster states, State 1 was characterized by the strongest correlation pattern, followed for correlation magnitude by State 3 and 4, which showed comparable strength, by State 2, and eventually by State 5 (figure 2). Two sample *t*-tests showed dFNC differences between EP and HC in 2 states (α < .05): in State 4, EP had an increased negative coupling between the EXE (IC26) and AUD (IC7); in State 5, characterized by the weakest correlation pattern, EP displayed reduced positive coupling within the SC (IC2 and IC4), reduced negative coupling between the SC (IC5) and the SM (IC8, IC9, and IC11), reduced positive coupling within the SM (IC8 and IC16) and reduced positive the SM (IC15) and the CB (IC51; supplementary figure 2). The cluster state analyses revealed that EP presented a longer dwelling in State 1 (*U* = 1208, *P* < .001), State 2 (*U* = 139, *P* < .001), and State 3 (*U* = 2020, *P* = .011), and reduced dwelling in State 4 (*U* = 1173, *P* < .001) and State 5 (*U* = 210, *P* < .001) compared to HC. Moreover, EP presented a higher NT (*U* = 2079, *P* = .02) (figure 3a, supplementary table 1). Meta-states analysis showed reduced state span (*U* = 1488, *P* < .001), number of states (*t* = −5.2, *P* < .001), number of state changes (*t* = −4.8, *P* < .001) and total distance (*t* = −4.58, *P* < .001) (figure 3b, supplementary table 1). The analyses on antipsychotic-free subjects confirm all the results (supplementary table 3). Results of dFNC analyses conducted with *k* = 4 and *k* = 6 are reported in supplementary material.

### Association Between Static and Dynamic Connectivity and Clinical and Cognitive Variables

**Fig. 4.**
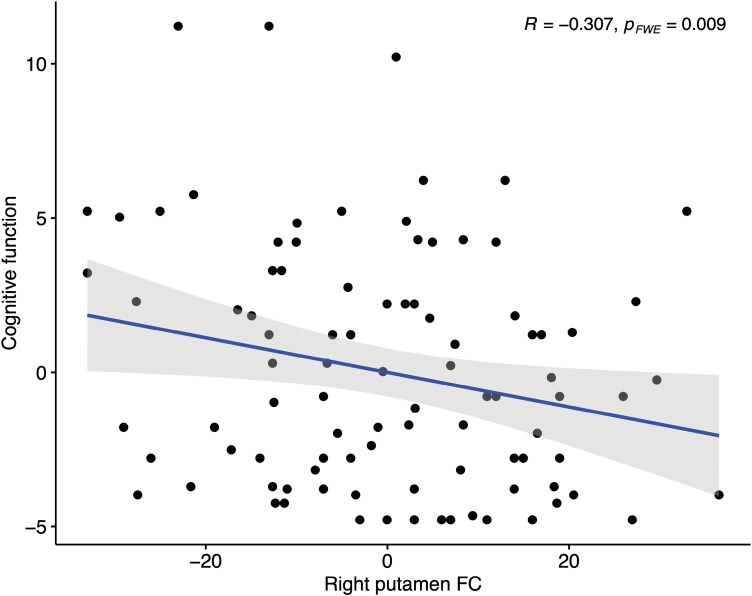
Scatterplot of the partial correlation between the NIH-T cognitive composite score, Box-Cox transformed and the sFC of the right striatum within SC, adjusted for chlorpromazine equivalents, correlation-adjusted Bonferroni corrected. FC, functional connectivity.

In EP, within the SC, the sFC of the right putamen (IC4) was negatively correlated with the NIH-T cognitive composite score (*r* = −0.326, *P* = .002, *p*_FWE_ = 0.009) (figure 4). At an uncorrected level, exploratory correlation analyses between sFC and psychopathology and cognition showed that, within the SM, the sFC of the left postcentral gyrus (IC16) and of the right SFG (IC10) was positively correlated with the PANSS positive score (rho = 0.250, *P* = .016 and rho = 0.225, *P* = .030), and the sFC of the left SFG (IC16) was positively correlated with the NIH-T cognitive composite score (rho = 0.216, *P* = .049); within the SC, the sFC of the right putamen (IC4) was positively correlated with the PANSS general score (*r* = 0.216, *P* = .042).

**Fig. 5.**
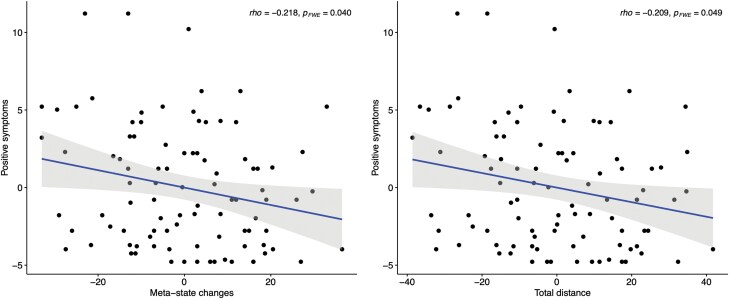
Scatterplots of partial correlations between PANSS positive scores and FC dynamism, adjusted for chlorpromazine equivalents, Bonferroni corrected.

As for dynamic connectivity, in the EP, PANSS positive score was negatively and significantly correlated with the number of meta-state changes and the total distance, applying a correlation-adjusted FWE correction (rho = −0.218, *p*_FWE_ = 0.040 and rho = −0.209, *p*_FWE_ = 0.049, see figure 5). No other significant correlations were found between the dFNC parameters and clinical or cognitive values in the exploratory analyses, even at an uncorrected level. The reassessment of significant correlations in patients without antipsychotics is reported in supplementary material.

## Discussion

The overarching objective of this study was to explore the intrinsic activity and the dynamics of the neural networks in a sample of EP and to examine the association between FC metrics and clinical and cognitive features. Three main findings emerged: (1) EP showed altered sFC in cortical and subcortical regions of the SC, SM, and VIS networks; (2) EP had reduced meta-state parameters, including the number of states, state changes, state span, and total distance of the meta-states compared to HC, as well as increased NT, longer dwell time in states characterized by high within-network correlation in SC and high anti-correlation between SC and the other networks, and shorter dwell time in the less connected state; (3) in EP, increased sFC of the right putamen within the SC network was associated with worse cognition and reduced dynamism was correlated with greater positive symptoms.

### Static Functional Network Connectivity

Compared to HC, EP showed an increase and a decrease in sFC in the striatum within the SC network. The striatum has been implicated in numerous high-order functions, including cognitive and emotion processing, goal-directed behaviors,^50^ reward anticipation,^51^ and regulation of impulsivity.^52^ Abnormalities in the ventral and dorsal striatum have been consistently reported in psychosis, regardless of disease stage, and also in individuals at genetic risk for the disorder.^53^ In particular, functional neuroimaging studies have described abnormal striatal coupling with default mode and CC networks in SCZ^54^ and significant FC alterations within the caudate nucleus in EP compared to HC.^55^ Moreover, recent meta-analytic evidence has shown that patients with EP present altered intrinsic neural activity in the striatum, and this was true also in antipsychotic-naïve individuals.^56^ The altered synchronization that we observed may be an effect of the increased mesostriatal dopaminergic firing, known to play a fundamental role in the pathogenesis of psychosis,^57,58^ and could be in relation to the dysconnectivity between striatum and other brain regions, that is a consistent finding in studies in EP.^2,14,59–62^ Notably, altered sFC of the striatum was correlated with cognitive function. This result corroborates previous studies that showed an association between structural and functional striatal alterations in SCZ and working memory deficits,^63^ abnormal attention processes,^64^ and furthermore, with negative symptoms.^65,66^ Several lines of research have suggested that, in psychosis, abnormalities in the subcortical DA system can affect the striatum, possibly resulting in cognitive deficits (see below).^58,67^

In addition, we also observed abnormalities in several regions of the SM network, including fronto-temporo-parietal areas, which is in line with the idea of psychomotor mechanisms in psychiatric disorders.^68^ Interestingly, alterations in the striatal and SM networks have also been demonstrated in first-episode drug-naïve SCZ patients, suggesting that altered dopaminergic function in the striatum might lead to abnormal FC within the subcortical-cortical circuitry.^69^ Notably, these authors also observed a correlation between thalamo-sensorimotor FC and psychopathological symptoms measured with the PANSS.^69^ Interestingly, our findings also corroborate a recent meta-analysis in schizophrenia that showed altered interconnections between the striatum and sensorimotor regions, including the lateral prefrontal cortex and pre-supplementary motor area.^70^ Taken together, this evidence suggests that striatal and SM systems are complexly linked and their interplay might be involved in the pathophysiology of psychotic disorders.

Lastly, EP presented lower sFC in the right precuneus and in the left SOG within the VIS network. Dysfunction in visual perception and in the visual system have long been associated with psychosis.^71–73^ In particular, alterations in VIS network FC have been reported both during task and at rest in SCZ, and these seem to be correlated with task-switching costs and with hallucination severity.^74^ In EP, a magnetoencephalography study demonstrated dysconnectivity across the VIS network in response to increasing cognitive demands, which was associated with positive symptoms.^75^ Interestingly, psychosis appears to be characterized by an aberrant peripheral visual signal, to which the thalamus and frontal cortex could first fail to adapt, followed by the lower visual processing areas.^76^ This mechanism be the basis of dysconnectivity within the VIS, along with perceptual and cognitive alterations.^76^

### dFNC

Our results showed that all of the dFNC parameters were altered in EP, including cluster states and meta-states. In general, EP had more occurrences of States 1 and 3, characterized by the strongest high within-network correlation in SC. Thus, the overall hyperconnectivity that we found within the SC network in sFC analyses might be linked to the longer time dwelled in these states. State 1 also presents the strongest anti-correlation between SC and AU, SM, and VIS. Similarly, State 3 is characterized by strong anti-correlation between SC and the same networks.

Previous studies in EP found a decrease in sFC between the striatum and several cortical areas related to auditory, sensorimotor, and visual functions.^61,77,78^ Differently, other studies reported an anteroposterior pattern with positive correlations in more frontal regions and negative correlations in more posterior regions.^15,79^ These results could reflect the effects of cortical dysfunction affecting dopaminergic or glutamatergic neurotransmission on striatal activity in psychosis,^64,80^ or a striatal indirect efference on cortical areas mediated by ventrotegmental area, substantia nigra, and thalamus.^64,67,80^

In line with prior research, the dFNC analysis revealed many FC alterations that were undetected by the sFC analysis, probably due to their time-varying occurrence. In EP, in the weakly connected State 5, the SM showed reduced negative coupling with the SC, in line with a previous finding of their hyperconnectivity in a sparsely connected state in psychotic patients,^28^ while reduced positive coupling with the CB and between its components. These alterations could contribute to explaining the presence of sensorimotor abnormalities described in treatment-naïve psychosis.^81,82^ In EP, the meta-state analysis showed a reduced number of states and lower total distance, which is consistent with earlier findings of reduced number of states, transitions, and limited state variability in SCZ^20,26^ Moreover, we found that EP had reduced state span and state changes. Recently, Ramirez-Mahaluf et al assessed the dFNC of EP clustering the whole brain configurations in many states to characterize the transitions between them. Patients showed longer dwelling within restricted groups of states and reduced shifts between very dissimilar states, which is highly consistent with our findings of reduced dynamic fluidity and range.^83^ In our study, the NT was higher in EP relative to HC. Previous evidence has shown an association between the NT and PANSS scores, suggesting a relationship between dFNC and symptom severity.^26^ This may suggest that patients with EP tend to have more frequent transitions between a limited number of states with frequent engagement and disengagement of networks with altered connectivity and this may probably be due to the instability deriving from the altered dopamine (DA) signaling, which is known to affect FC.^84,85^ Moreover, reduced dynamic fluidity and increased persistence in states with disrupted FC in EP can indicate a reduced temporal coordination between brain networks implicated in affect, motor function, and behavior.^86^ These changes may be reflected by an overall impairment of the dynamics of the brain, not only at the level of specific states, where EP tend to dwell more in states with increased network interplay, but also at a global level with reduced overall dynamics of the brain, as previously shown in SCZ and BD.^87,88^

Consistently with our hypothesis and with the previous finding by Rabany et al,^26^ abnormalities in dynamic fluidity and range were associated with positive symptoms. Notably, this association was not driven by age or antipsychotic treatment. Notably, our findings in EP are in partial contrast with the findings of Miller et al in chronic SCZ, in which hallucinations were negatively correlated with dynamic fluidity, while delusions were weakly positively correlated.^20^ We speculate that the latter results might be due to the confounding effects of the chronicity of the disorder and the treatment, and lifestyle changes that characterize patients with SCZ and support the rationale of investigating dysconnectivity in EP.

### Dopamine Alterations and Functional Dysconnectivity

Dopamine dysregulation has a central role in psychosis’ neurobiology, and multiple evidence links it to the rising of both cognitive and positive symptoms,^89^ as well as to FC aberrancies,^90,91^ although the specific mechanisms are still unknown. The DA levels are known to have an inverted U-shaped relationship with cognitive functions, such as working memory and cognitive control,^92,93^ while the relationship with brain activity and FC appears to be more complex.^92,94,95^ For instance, Tian et al found that the estimated DA levels had an inverted U-shaped relationship with the FC of the prefrontal cortex, while an upright U-shaped relationship with the FC of the striatum.^94^ Hypothetically, the striatal FC increase that we observed can follow from increased striatal DA signaling, which could result in cognitive deficits via altered cortico-striato-thalamic circuits.^64,67^

DA levels could also impact the dFNC, as suggested by the effect of antidopaminergic treatments on the time dwelled in different dynamic states.^96,97^ Moreover, the DA activity, which is characterized by a complex balance of tonic and phasic bursts,^98^ could influence the dynamic fluidity and range. Interestingly, a major hypothesis on psychosis is that the steady-state tonic bursts are reduced, determining an increase of DA, thus increasing the phasic bursts, produced by external stimuli, and leading to symptoms.^98^

### Strengths and Limitations

The sample size, detailed clinical and cognitive assessment, and the use of a comprehensive set of sophisticated FC measures represent the strengths of our study. However, some methodological aspects limit the generalizability of our results. First, the patients were not antipsychotic-naïve, and, although to a lesser extent than patients with chronic exposition, their FC could be influenced by medications. Nonetheless, we used statistical correction for this nuisance variable and repeated our analyses in a drug-free sample, and confirmed our main findings. Second, although psychosis could be conceptualized as a syndrome associated with different illnesses, combining data from non-affective and affective disorders could have hidden some differences in the biology of psychosis in these distinct disorders.^99^ Nonetheless, we wanted to study alterations of brain connectivity that were shared across disorders and underlie a common construct of psychosis. Third, we imposed the number of clusters (*k* = 5) in accordance with previous literature. We also experimented by varying *k* between 4 and 6, but the results showed reduced sensitivity to group differences in their temporal dynamics. An important future direction for dFNC studies will be to motivate optimal choice of the number of clusters, as well as clustering methods. Lastly, we did not validate our results in an external dataset.

## Conclusions

In conclusion, by using static and dynamic FC analyses focusing on the changes of the brain network configurations over time, we provided further evidence of dysconnectivity in EP. In particular, we observed static hyperconnectivity of the bilateral striatum in the SC network, converging with the dopaminergic hypothesis of psychosis. Additionally, we displayed lower dynamism in EP compared to HC, suggesting a reduced temporal coordination between brain networks implicated in affect, motor function, and behavior. Moreover, in EP, we detected a negative correlation between fluid and crystallized cognitive functions and the sFC of the right striatum and a negative correlation between positive symptoms and dynamism. If replicated, these results may inform future research to design neural intervention strategies targeted at developing novel effective treatments.

## Supplementary Material

Supplementary material is available at https://academic.oup.com/schizophreniabulletin/.

sbae142_suppl_Supplementary_Material
