# Supplementary material for: Static and Dynamic Dysconnectivity in Early Psychosis: Relationship With Symptom Dimensions
Source: Schizophr Bull. 2024 Aug 30;51(1):120–32. doi: 10.1093/schbul/sbae142 (PMC11661956; doi:10.1093/schbul/sbae142)

**Static and dynamic dysconnectivity in early episode psychosis: Relationship with symptom dimensions**

*Running title:* Brain dysconnectivity in early psychosis

*Supplementary Material*

Contents:

Supplementary Results

Supplementary Tables S.1-S.7

Supplementary Figures S.1-S.8

**Analyses excluding the antipsychotic-treated subjects**

All the between-group differences found between early psychosis (EP) and healthy controls (HC) were significant excluding the antipsychotic-treated subjects, thus comparing 52 antipsychotic-free EP and 56 HC (Tables S.3-S.5). The significant partial correlations were reassessed in the antipsychotic-free group using one-sided Pearson’s and Spearman’s correlations as appropriate. The negative correlation between PANSS positive score and the meta-state changes showed rho=-0.251 and p=0.038, while the one between PANSS positive and the total distance showed rho=-0.229 and p=0.053. The negative correlation between the NIHT cognitive composite score, boxcox transformed and the right putaminal FC (IC4) displayed r=-0.411 and p=0.003.

**Table S.1. Dynamic FC parameters in EP and HC**

|  | **EP**  **(n=96)**  **Mean (SEM)** | **HC**  **(n=56)**  **Mean (SEM)** | **t or U** | **p** |
| --- | --- | --- | --- | --- |
| **Dwell State 1** | 20.99 (1.53) | 7.93 (1.37) | U=1208 | <0.001 |
| **Dwell State 2** | 47.97 (4.85) | 3.24 (0.87) | U=139 | <0.001 |
| **Dwell State 3** | 21.40 (1.71) | 14.90 (1.45) | U=2020 | 0.011 |
| **Dwell State 4** | 3.90 (1.51) | 33.63 (5.63) | U=1173 | <0.001 |
| **Dwell State 5** | 6.12 (0.93) | 50.93 (6.91) | U=210 | <0.001 |
| **N. transitions cluster** | 11.54 (0.52) | 9.50 (0.58) | U=2079 | 0.02 |
| **N. states** | 24.76 (1.12) | 34.00 (1.31) | t=-5.2 | <0.001 |
| **Change states** | 52.74 (1.64) | 65.32 (1.97) | t=-4.8 | <0.001 |
| **State span** | 7.68 (0.22) | 9.18 (0.16) | U=1488 | <0.001 |
| **Total distance** | 57.90 (1.95) | 72.13 (2.29) | t=-4.58 | <0.001 |

EP: early psychosis; HC: healthy controls; SEM: standard error of mean

**Table S.2. Head motion parameters in EP compared to HC**

|  | **EP**  **(n=52)** | **HC**  **(n=56)** | **t or U** | **p** |
| --- | --- | --- | --- | --- |
| **Mean RMS** | 0.327 ± 0.223 | 0.301 ± 0.199 | U=2455 | 0.347 |
| **Mean FD Power** | 0.187 ± 0.0777 | 0.158 ± 0.0406 | U=2140 | 0.036 |

EP: early psychosis; FD: framewise displacement; HC: healthy controls; RMS: root mean square

**Table S.3. Demographic and clinical variables excluding the antipsychotic treated subjects**

|  | **AP-free EP**  **(n=52)** | **HC**  **(n=56)** | *χ*^2^**or t** | **p** |
| --- | --- | --- | --- | --- |
| **Age [m (SD), ys]** | 22.58±3.93 | 24.75±4.15 | U=983 | 0.004 |
| **Sex [M/F]** | 29/23 | 37/19 | *χ*^2^=1.20 | 0.272 |
| **Handedness (R/L/A)** | 46/5/1 | 45/10/1 | *χ*^2^=1.73 | 0.421 |
| **Full IQ** | 106±15.5 | 116±10.6 | U=849 | <0.001 |
| **SES** | 1.85± 0.92 | 2.09 ±1.06 | U=1245 | 0.219 |
| **AP exposure (months)** | 12.90±15.71 | - | - | - |

A: ambidextrous; AP: antipsychotics; EP: early psychosis; F: female; HC: healthy controls; IQ: intelligence quotient; L: left; m: mean; M: male; R: right; SD: standard deviation; SES: socio-economic status

**Table S.4. Static FC parameters in antipsychotic-free EP compared to HC**

|  | **EP**  **(n=52)** | **HC**  **(n=56)** | **t or U** | **p** |
| --- | --- | --- | --- | --- |
| Left striatum | 3.87 ± 0.61 | 3.33 ± 0.62 | t=4.57 | <0.001 |
| Right striatum | 5.49 ± 0.85 | 5.09 ± 0.69 | Welch’s t=2.66 | 0.009 |
| Right striatum | 3.62 ± 0.63 | 4.05 ± 0.67 | t=-3.36 | 0.001 |
| Left STG | 1.26 ± 0.81 | 0.75 ± 0.73 | U=946 | 0.002 |
| Right STG | 0.48 ± 0.61 | 0.04 ± 0.41 | Welch’s t=4.40 | <0.001 |
| Right SFG | 0.75 ± 0.75 | 1.36 ± 0.77 | t=-4.14 | <0.001 |
| Left postcentral gyrus | 2.07 ± 1.24 | 2.92 ± 1.40 | t=-3.30 | 0.001 |
| Right precentral gyrus | 8.11 ± 2.09 | 9.63 ± 1.94 | U=817 | <0.001 |
| Right precuneus | 4.32 ± 1.62 | 5.68 ± 1.50 | U=762 | <0.001 |
| Left SFG | 1.04 ± 0.98 | 0.53 ± 0.60 | U=917 | <0.001 |
| Left IPG | 2.89 ± 1.22 | 2.09 ± 0.69 | U=843 | <0.001 |
| Right postcentral gyrus | 3.53 ± 1.44 | 3.00 ± 1.17 | U=1129 | 0.045 |
| Left postcentral gyrus | 2.09 ± 1.43 | 3.14 ± 1.61 | U=1677 | <0.001 |
| Right precentral gyrus | 1.69 ± 1.32 | 2.78 ± 1.35 | U=942 | 0.002 |
| Right precuneus | 4.83 ± 1.41 | 5.58 ± 1.71 | t=-2.48 | 0.015 |
| Left SOG | 1.71 ± 0.98 | 2.24 ± 1.21 | t=-2.52 | 0.013 |

EP: early psychosis; HC: healthy controls; IPG: superior parietal gyrus; SFG: superior frontal gyrus; SOG: superior occipital gyrus; STG: superior temporal gyrus

**Table S.5. Dynamic FC parameters in antipsychotic-free EP compared to HC**

|  | **EP**  **(n=52)** | **HC**  **(n=56)** | **t or U** | **p** |
| --- | --- | --- | --- | --- |
| **Dwell State 1** | 17.37 ± 11.65 | 7.93 ± 10.29 | U=738 | <0.001 |
| **Dwell State 2** | 51.47 ± 39.01 | 3.24 ± 6.53 | U=65 | <0.001 |
| **Dwell State 3** | 20.92 ± 13.41 | 14.90 ± 10.88 | U=1057 | 0.014 |
| **Dwell State 4** | 3.26 ± 8.71 | 33.63 ± 42.18 | U=651 | <0.001 |
| **Dwell State 5** | 6.88 ± 9.03 | 50.93 ± 51.71 | U=115 | <0.001 |
| **N. transitions cluster** | 11.48 ± 5.37 | 9.50 ± 4.40 | t=2.10 | 0.038 |
| **N. states** | 25.71 ± 10.89 | 34.00 ± 9.77 | t=-4.17 | <0.001 |
| **Change states** | 54.21 ± 15.60 | 65.32 ± 14.78 | U=844 | <0.001 |
| **State span** | 7.87 ± 2.21 | 9.18 ± 1.25 | Welch’s t=-3.76 | <0.001 |
| **Total distance** | 60.00 ± 18.93 | 72.13 ± 17.07 | t=-3.50 | <0.001 |

EP: early psychosis; HC: healthy controls

**Table S.6. Dynamic FC parameters in EP and HC.** The cluster states resulting from the dFNC analysis were identified by k-means clustering (k=4) at the whole-group level.

|  | **EP**  **(n=96)**  **Mean (SEM)** | **HC**  **(n=56)**  **Mean (SEM)** | **t or U** | **p** |
| --- | --- | --- | --- | --- |
| **Dwell State 1** | 23.2 (1.66) | 19.1 (1.44) | U=2303 | 0.142 |
| **Dwell State 2** | 26.0 (2.31) | 13.9 (1.60) | U=1568 | <0.001 |
| **Dwell State 3** | 8.38 (1.78) | 33.3 (2.78) | U=1702 | <0.001 |
| **Dwell State 4** | 29.9 (1.79) | 32.5 (2.83) | U=2450 | 0.364 |
| **N. transitions cluster** | 12.8 (0.44) | 12.0 (0.60) | t=-1.06 | 0.289 |
| **N. states** | 22.2 (0.90) | 26.2 (1.25) | U=2055 | 0.016 |
| **Change states** | 53.9 (1.40) | 56.8 (1.99) | t=1.19 | 0.237 |
| **State span** | 7.58 (0.19) | 8.25 (0.22) | U=2157 | 0.033 |
| **Total distance** | 58.3 (1.61) | 62.1 (2.22) | t=1.41 | 0.161 |

EP: early psychosis; HC: healthy controls; SEM: standard error of mean

**Table S.7. Dynamic FC parameters in EP and HC.** The cluster states resulting from the dFNC analysis were identified by k-means clustering (k=6) at the whole-group level.

|  | **EP**  **(n=96)**  **Mean (SEM)** | **HC**  **(n=56)**  **Mean (SEM)** | **t or U** | **p** |
| --- | --- | --- | --- | --- |
| **Dwell State 1** | 18.2 (1.57) | 15.3 (1.89) | U=2382 | 0.241 |
| **Dwell State 2** | 22.2 (1.50) | 25.8 (2.88) | U=2521 | 0.524 |
| **Dwell State 3** | 20.1 (1.59) | 11.6 (1.16) | U=1732 | <0.001 |
| **Dwell State 4** | 14.3 (1.29) | 18.0 (1.60) | U=2169 | 0.047 |
| **Dwell State 5** | 18.9 (1.26) | 16.7 (1.45) | U=2394 | 0.261 |
| **Dwell State 6** | 4.67 (0.39) | 20.6 (2.51) | U=1828 | <0.001 |
| **N. transitions cluster** | 15.5 (0.44) | 14.5 (0.67) | t=2312 | 0.150 |
| **N. states** | 59.4 (1.77) | 62.5 (1.96) | Welch’s t=1.135 | 0.258 |
| **Change states** | 85.3 (1.61) | 86.7 (1.94) | t=0.532 | 0.595 |
| **State span** | 11.6 (0.23) | 12.4 (0.26) | U=2136 | 0.033 |
| **Total distance** | 98.3 (2.14) | 101 (2.58) | t=0.940 | 0.349 |

EP: early psychosis; HC: healthy controls; SEM: standard error of mean

**Fig. S.1.** Design matrix showing the effect of predictors (age, diagnosis, mean frame-wise displacement, and root mean square of motion) on the MANCOVA on the spatial maps during resting state. Intrinsic Networks (Ins) are grouped by type of network.

**
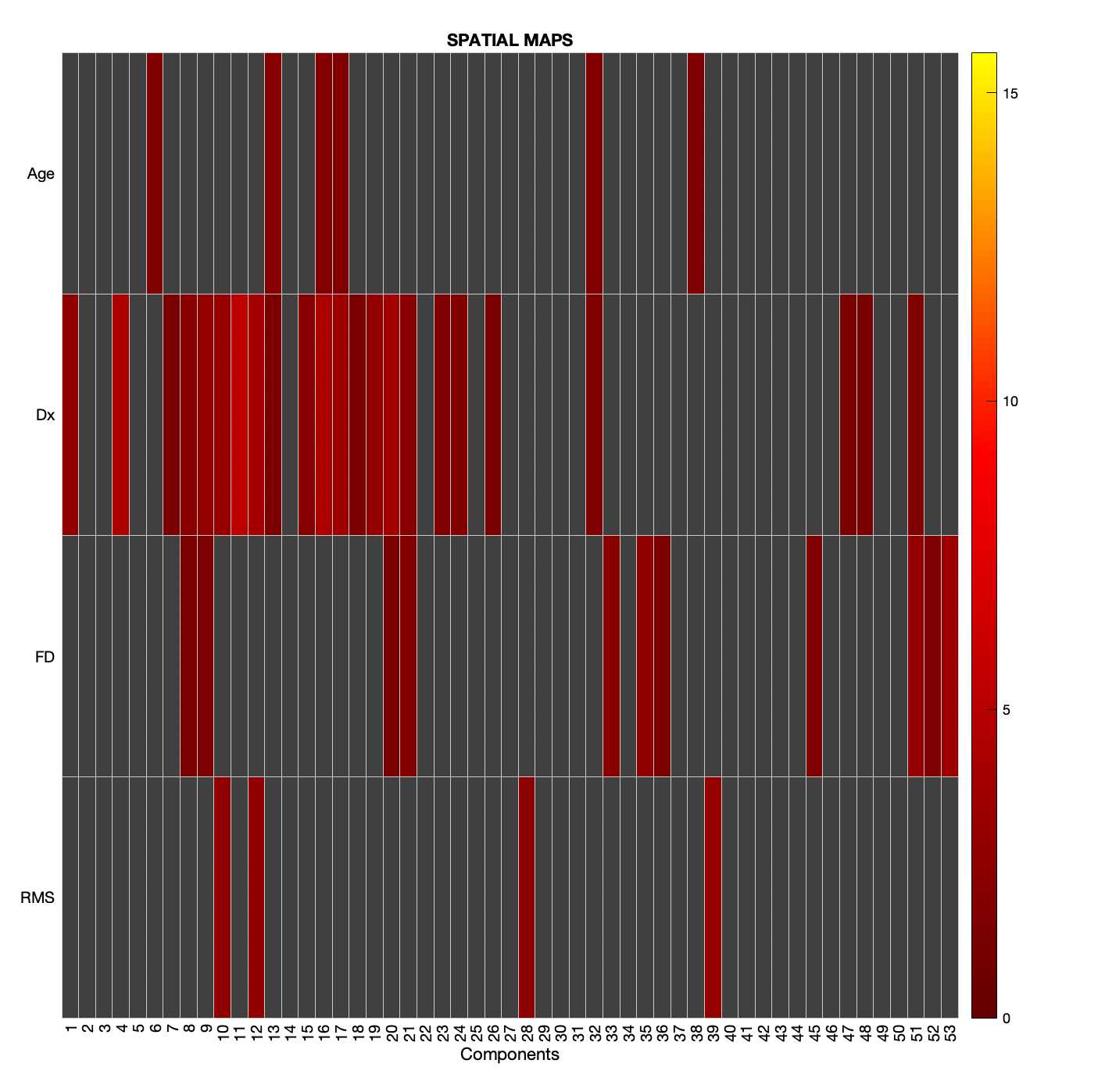
**

**Fig. S.2.** Two sample t-tests showed dynamic functional network connectivity (dFNC) differences between the first episode psychosis individuals and healthy controls in State 4 and State 5. The cluster states were identified by k-means clustering (k=5) at the whole-group level. The color bar indicates the magnitude of each correlation.

**
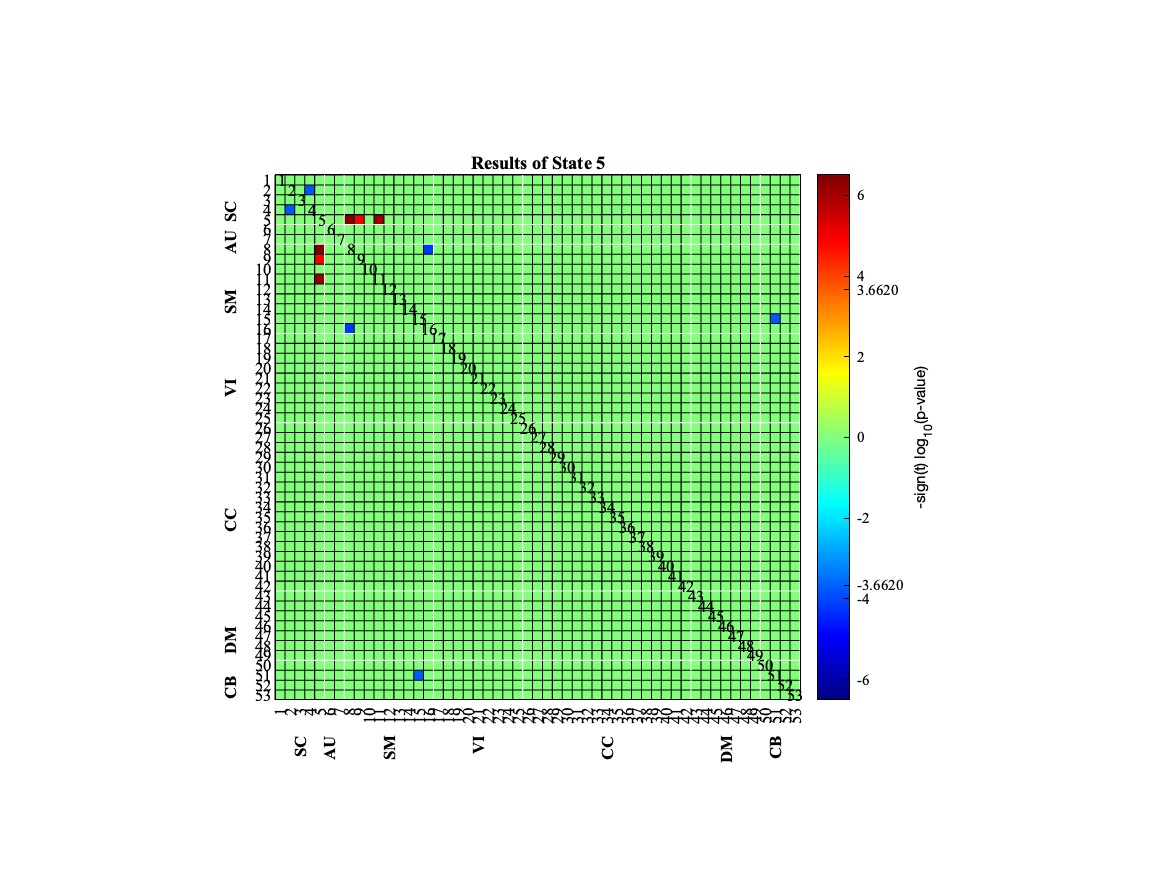

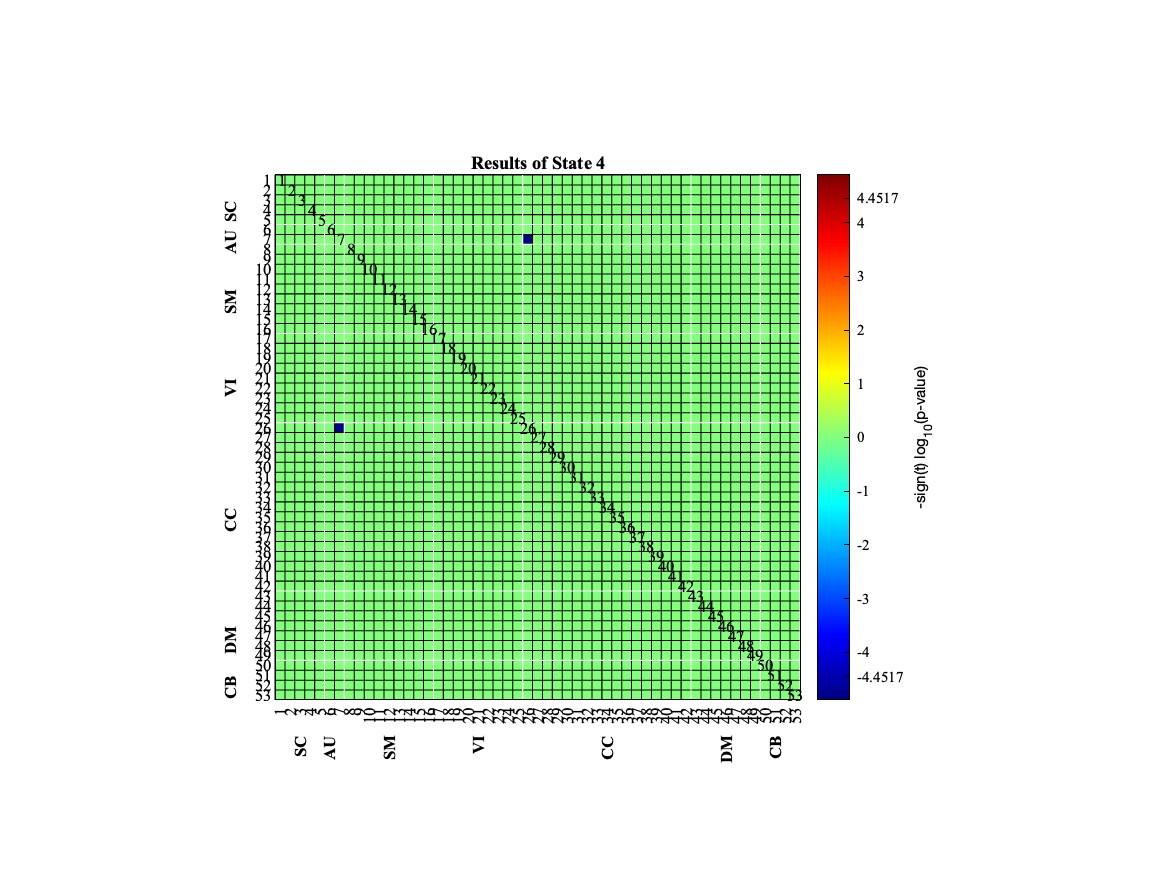
**

**Fig. S.3.** The cluster states resulting from the dFNC analysis were identified by k-means clustering (k=4) at the whole-group level. The median cluster centroids are reported in the correlation matrices for each of the five dFNC states. The color bar indicates the magnitude of each correlation. SC: subcortical network; AU: auditory network; SM: sensorimotor network; VI: visual network; CC: cognitive control network; DM: default mode network; CB: cerebellum network.

**Fig. S.4.** Two sample t-tests showed dynamic functional network connectivity (dFNC) differences between the first episode psychosis individuals and healthy controls in State 2, State 4, and State 5. The cluster states were identified by k-means clustering (k=4) at the whole-group level. The color bar indicates the magnitude of each correlation.


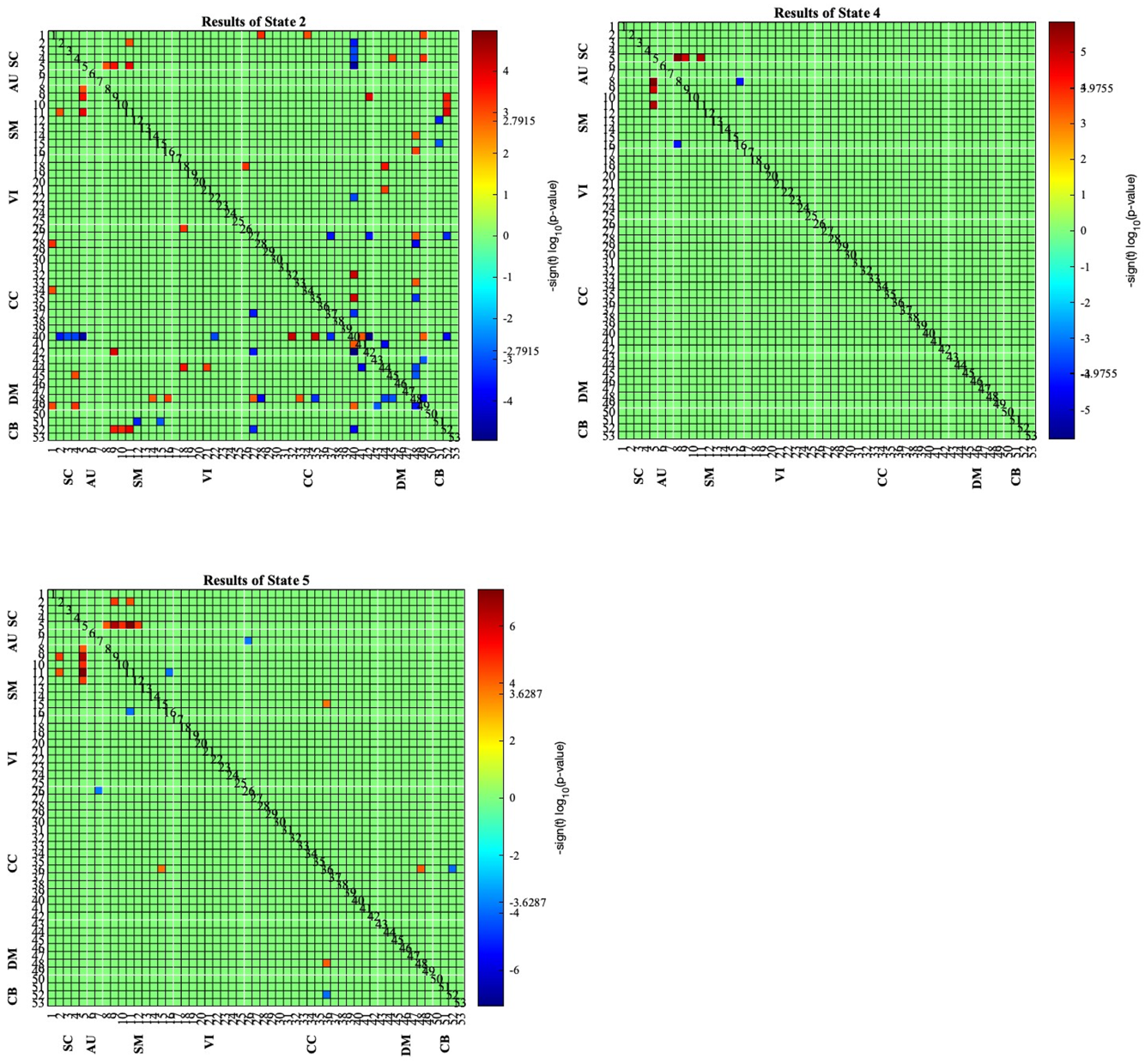


**Fig. S.5. a)** Dwell times in the different state clusters and number of transitions between clusters in EP and HC with respective standard error. EP is indicated in blue, and HC in orange. Dwell times are given as the number of TR windows. All the differences are significant at p < .05. NT: number of transitions. **b)** Number of meta-states, number of state changes, state span, and total distance, with respective confidence intervals. The cluster states resulting from the dFNC analysis were identified by k-means clustering (k=4) at the whole-group level. All the differences are significant at p < .05.


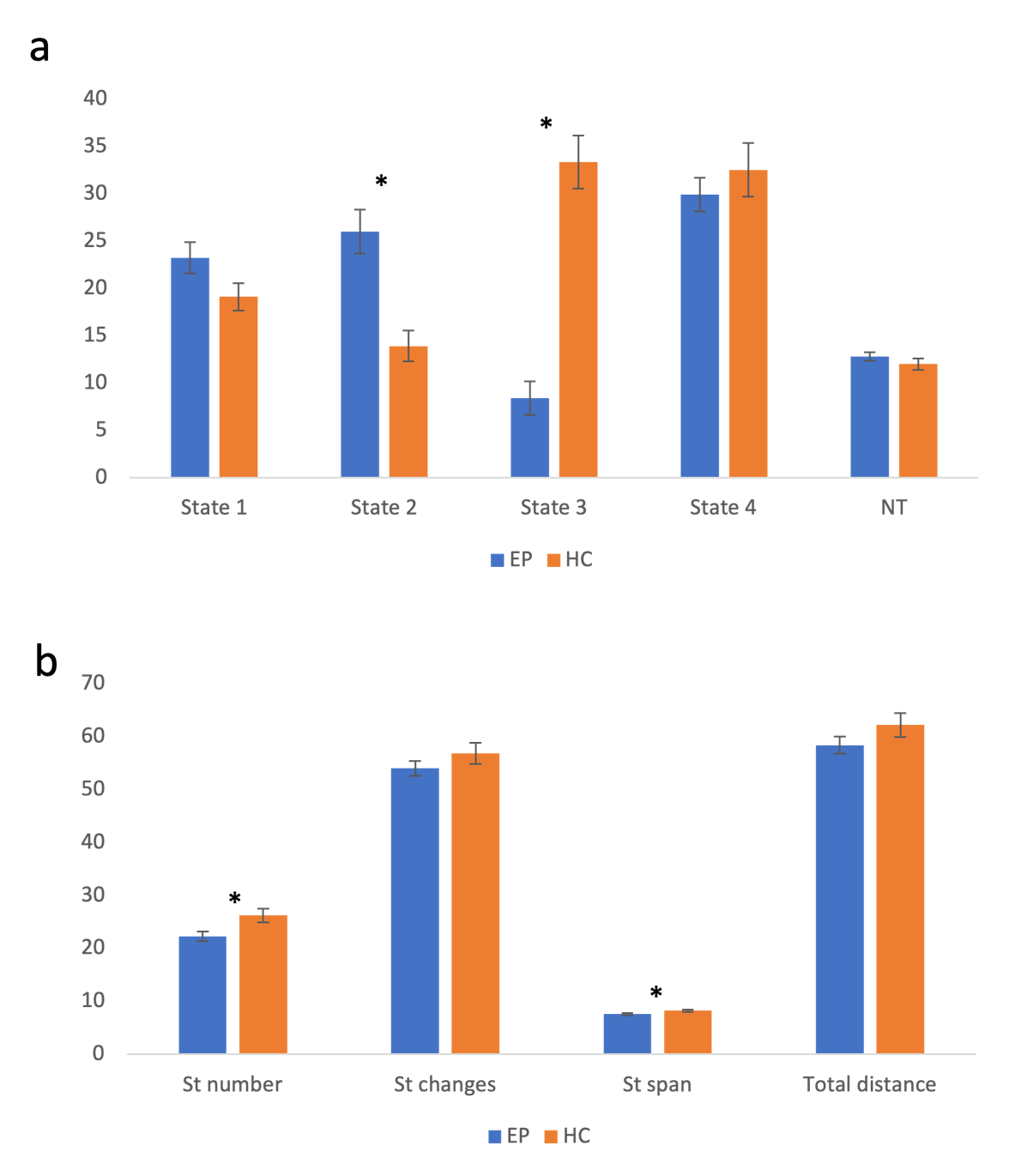


**Fig.S.6.** The cluster states resulting from the dFNC analysis were identified by k-means clustering (k=6) at the whole-group level. The median cluster centroids are reported in the correlation matrices for each of the five dFNC states. The color bar indicates the magnitude of each correlation. SC: subcortical network; AU: auditory network; SM: sensorimotor network; VI: visual network; CC: cognitive control network; DM: default mode network; CB: cerebellum network.

**Fig. S.7.** Two sample t-tests showed dynamic functional network connectivity (dFNC) differences between the first episode psychosis individuals and healthy controls in State 2 and State 5. The cluster states were identified by k-means clustering (k=6) at the whole-group level. The color bar indicates the magnitude of each correlation.


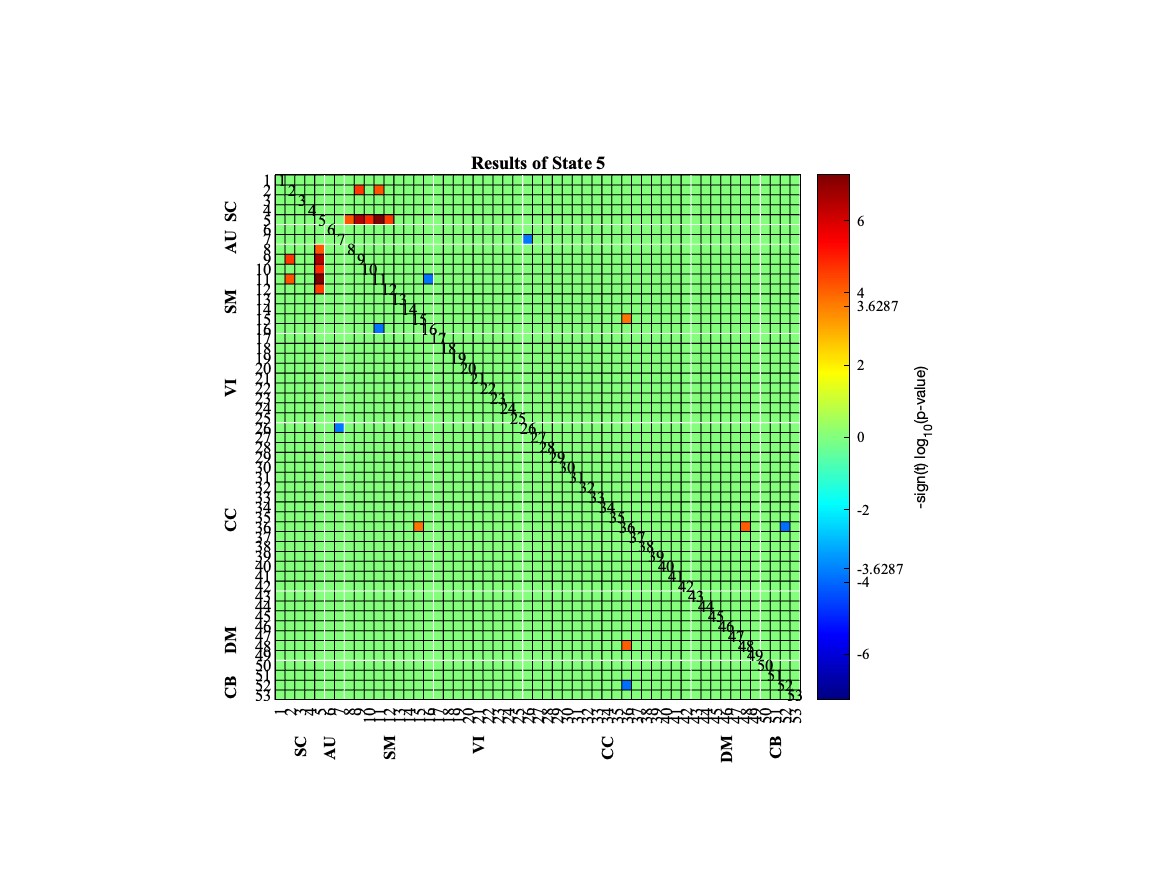

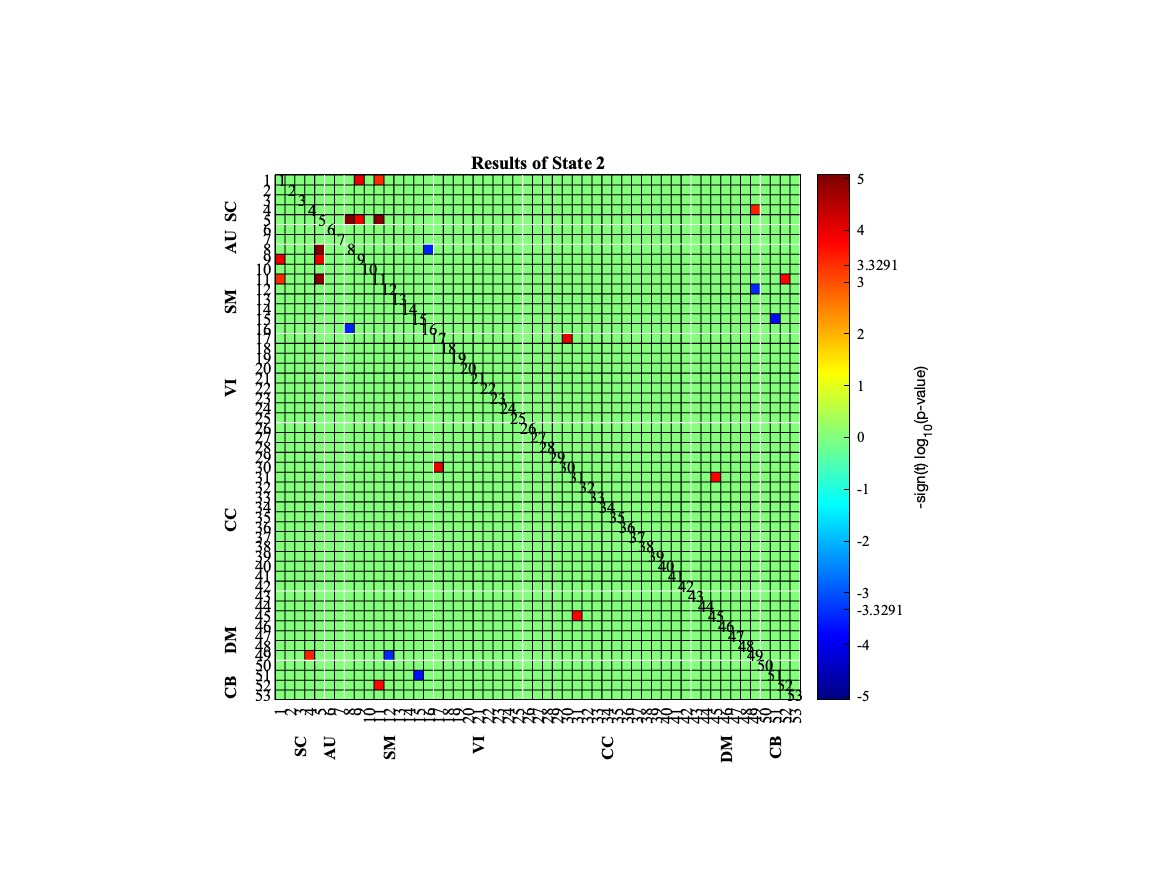


**Fig. S.8. a)** Dwell times in the different state clusters and number of transitions between clusters in EP and HC with respective standard error. EP is indicated in blue, and HC in orange. Dwell times are given as the number of TR windows. All the differences are significant at p < .05. NT: number of transitions. **b)** Number of meta-states, number of state changes, state span, and total distance, with respective confidence intervals. The cluster states resulting from the dFNC analysis were identified by k-means clustering (k=6) at the whole-group level. All the differences are significant at p < .05.


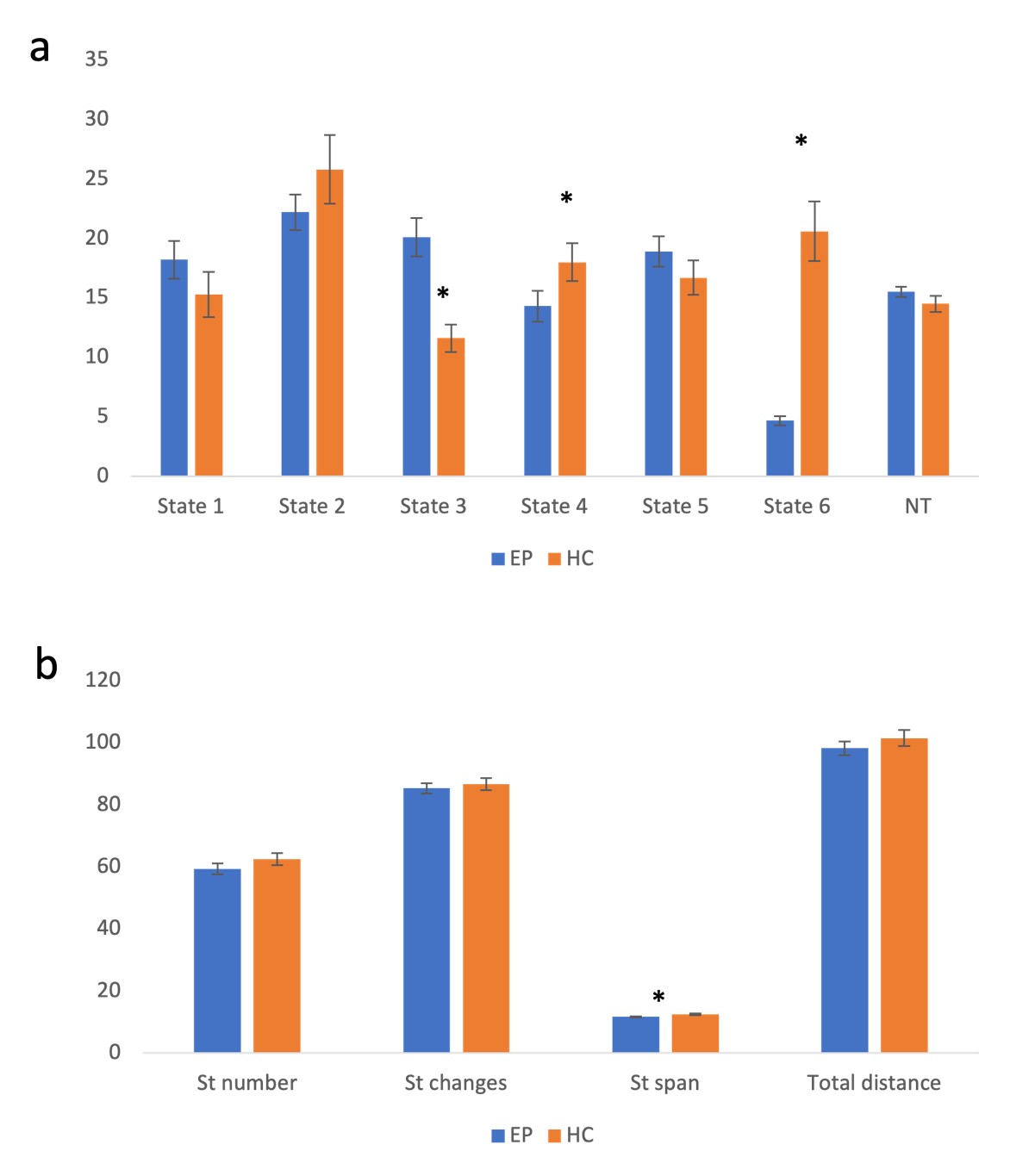

Supplement: sbae142_suppl_Supplementary_Material [file sbae142_suppl_supplementary_material.docx]
